# Validation of MuLBSTA score to derive modified MuLB score as mortality risk prediction in COVID-19 infection

**DOI:** 10.1371/journal.pgph.0000511

**Published:** 2022-08-01

**Authors:** Richie George, Asmita A. Mehta, Tisa Paul, Dipu T. Sathyapalan, Nithya Haridas, Akhilesh Kunoor, Greeshma C. Ravindran

**Affiliations:** 1 Department of Respiratory Medicine, Amrita Institute of Medical Sciences, Amrita Vishwa Vidyapeetham, Kochi, Kerala, India; 2 Division of infectious Diseases, Department of Internal Medicine, Amrita Institute of Medical Sciences, Amrita Vishwa Vidyapeetham, Kochi, Kerala, India; 3 Department of Biostatistics, Amrita Institute of Medical Sciences, Amrita Vishwa Vidyapeetham, Kochi, Kerala, India; Public Health Ontario, CANADA

## Abstract

COVID-19pandemic was started in December 2019. It has variable presentation from mild sore throat to severe respiratory distress. It is important to identify individuals who are likely to worsen. The Research question is how to identify patients with COVID-19 who are at high risk and to predict patient outcome based on a risk stratification model? We evaluated 251 patients with COVID-19 in this prospective inception study. We used a multi-variable Cox proportional hazards model to identify the independent prognostic risk factors and created a risk score model on the basis of available MuLBSTA score. The model was validated in an independent group of patients from October2020 to December 2021. We developed a combined risk score, the MuLBA score that included the following values and scores: Multi lobar infiltrates (negative0.254, 2), lymphopenia (lymphocytes of <0.8x10^9^ /L, negative0.18,2), bacterial co- infection (negative, 0.306,3). In our MuLB scoring system, score of >8 was associated with high risk of mortality and <5 was at mild risk of mortality (P < 0.001). The interpretation was that The MuLB risk score model could help to predict survival in patients with severe COVID-19 infection and to guide further clinical research on risk-based treatment.

## Introduction

The novel corona virus disease 2019 (COVID-19) has affected the public across the globe and continue to be an important and urgent threat to global health. Since the outbreak in early December 2019 in the Hubei province of the People’s Republic of China, the number of patients confirmed to have the disease has exceeded 5,507,376 in more than 160 countries, and the number of people infected is 308,034,154 as of 10-1-22 [1] Efficient diagnosis and better predictors of prognosis in COVID-19 are needed to mitigate the burden on the healthcare system [2]. Prediction models comprising of clinical features or laboratory parameters that can estimate the risk of people poor outcome from the COVID-19 infection could assist medical staff to triage patients when allocating limited healthcare resources [3]. A variety of clinical prediction scores for community acquire pneumonia such as CURB-65 and PSI are widely used in the assessment [4], they remain not applicable in the setting of viral infection. There are other reported risk factors such as PO2/FiO2, lymphocyte count, and antigen-specific T cells used for predicting mortality and deciding on appropriate for influenza pneumonia. (Viasus et al., 2016; Shi et al., 2017). Various models ranging from rule based scoring systems to advanced earning models have been proposed and published that are relevant for COVID-19 infection and have helped to save lives [3–7].

The “Multi-lobar infiltration, hypo lymphocytosis, bacterial co-infection, smoking history, hyper-tension and age score” abbreviated as the MuLBSTA score is one such proposed model which is likely to assist medical professional in arriving at a clinical decision [5]. The researchers Guo L, Wei D et al. was able to demonstrate that the MuLBSTA score could help in predicting the ninety-day mortality in viral pneumonia [6]. MuLBSTA scoring system was proven to be a good model for predicting mortality and risk stratification of the patients in a study by Rong Xu et al. Prognosis scores routinely used for CAP (PSI and CURB-65) were good predictors for mortality in patients with COVID-19 CAP but not for need of hospitalization or ICU admission. Study by Garcia Clement MM. et al. showed that MuLBSTA score was better at predicting the need for ICU admissions than other prognostic scores such as PSI‑PORT and CURB‑65 [7].

This study was aimed at validating MuLBSTA score [3, 4] in predicting 28- day mortality and prognosis in seriously ill COVID-19 patients.

### Aims and objectives

Primary objective of the current study was to identify and validate MuLBSTA score as clinical indicator tool that could predict the prognosis of patients with severe COVID-19 during admission in the study cohort. We also aimed to establish and validate MulBSTA score and to derive modified score if possible.

Secondary objective of the study was to identify other prognostic markers by studying the association of various laboratory parameters and the comorbidities with mortality in severe COVID 19 infection in a tertiary setting.

### Study methodology

This was an analytical cohort study to validate the role of MuLBSTA score in estimating 28-day mortality. The study was commenced after receiving an authorization certificate from both the scientific and ethical committee of Amrita Institute of Medical Sciences and Research Centre, Kochi. Informed written consent was taken from all patients before enrolling them in the study. The study included analysis of the clinical characteristics and laboratory parameters during the time of hospitalization between the survivor and the non-survivor groups. Patients admitted with severe COVID 19 infection between October 2020 and December 2021 were considered in our study. Based on the proportion of greater than or equal to 12 of MuLBSTA score in alive patients (3.6%) and odds ratio of 22.29 observed in an earlier study on “MuLBSTA score in COVID-19 pneumonia and prediction of 14-day mortality risk “by Preetham and et al. [5] and with 80% power and 95% confidence the least sample size is fourteen each (survivors as well as non-survivors).

Study definitions: [8]

COVID-19 illness was categorized as mild, moderate or severe as per following symptomatology.

Mild COVID-19 Illness: Individuals who have any of the various signs and symptoms of COVID-19 (e.g., fever, cough, sore throat, malaise, headache, muscle pain, nausea, vomiting, diarrhoea, loss of taste and smell) but who do not have shortness of breath, dyspnoea, or abnormal chest imaging.

Moderate COVID-19 Illness: Individuals who show evidence of lower respiratory disease during clinical assessment or imaging and who have an oxygen saturation (SpO_2_) ≥94% on room air at sea level.

- **Severe COVID-**19 was defined as Individuals with SpO_2_ <94% on room air at sea level, a ratio of arterial partial pressure of oxygen to fraction of inspired oxygen (PaO_2_/FiO_2_) <300 mm Hg, a respiratory rate >30 breaths/min, or lung infiltrates >50%.
- **Inclusion criteria**- All consecutive patients admitted with severe COVID-19 infection from October 2020 and December 2021 were included. All patients with diagnosis of COVID-19 with RT PCR/antigen/Xpert who required admission either in high dependency unit (HDU) or intensive care unit were included in the study.
- **Exclusion criteria**: Patients <18 yrs. of age, 2) explicit refusal to participate in the study and 3) patients with mild or moderate COVID-19 infection that did not require admission in ICU or HDU were excluded.

Demographics characteristics, history, clinical presentation laboratory findings, and radiological features were noted. The MuLBSTA score was calculated as showed in S1 Table [5, 6], and the patients were classified into survivors and non-survivor’s group. The MuLBSTA score and its association with mortality at 28 days thus determining its use as a prognostic tool was studied. Individual clinical parameters like Absolute lymphocyte count, Serum Albumin, Serum sodium, Platelet counts, Serum lactate dehydrogenase levels, Serum Ferritin, C reactive protein, D Dimer were taken into account along with the presence of co-morbidities like type 2 Diabetes mellitus (DM), chronic liver disease (CLD), chronic kidney disease (CKD), coronary artery disease (CAD), cerebrovascular accident (CVA), obstructive airway disease (OAD), hypothyroidism, obstructive sleep apnea (OSA) and its value as a prognostic marker was studied. The standard cut offs were applied as per the guidelines published by the Government of Kerala in managing the COVID pandemic as of August 2021 [7].

### Statistical analysis

Statistical analysis was done using IBM SPSS 20. (SPSS Inc, Chicago, USA). For all the continuous variables, the results are given in Mean ± SD, and for categorical variables as percentage. We used the x^2^ test or Fisher exact test to compare the categorical variables between different groups and the Mann-Whitney U test to compare median differences between the two groups for continuous variables. Uni variate and multivariate Cox regression models served as the main statistical methods for identification of prognostic factors for 28-day mortality. All the variables found to have P value of <0.05 in Uni variate analysis were subjected to multivariate analysis. We used a multi variable logistic regression analysis model to identify independent prognostic risk factors and calculate their HR’s, 95% CI, and beta regression coefficients. To estimate the overall survival time of samples, Kaplan Meier curves was used. The optimal cut-off value for MuLBSTA score was determined with the use of receiver operating characteristic curve analysis, then each continuous parameter was converted into a classification variable. For all analyses, statistical significance was calculated with the use of two-tailed probability values; P < .05 was considered significant.

## Results

**Fig 1:**
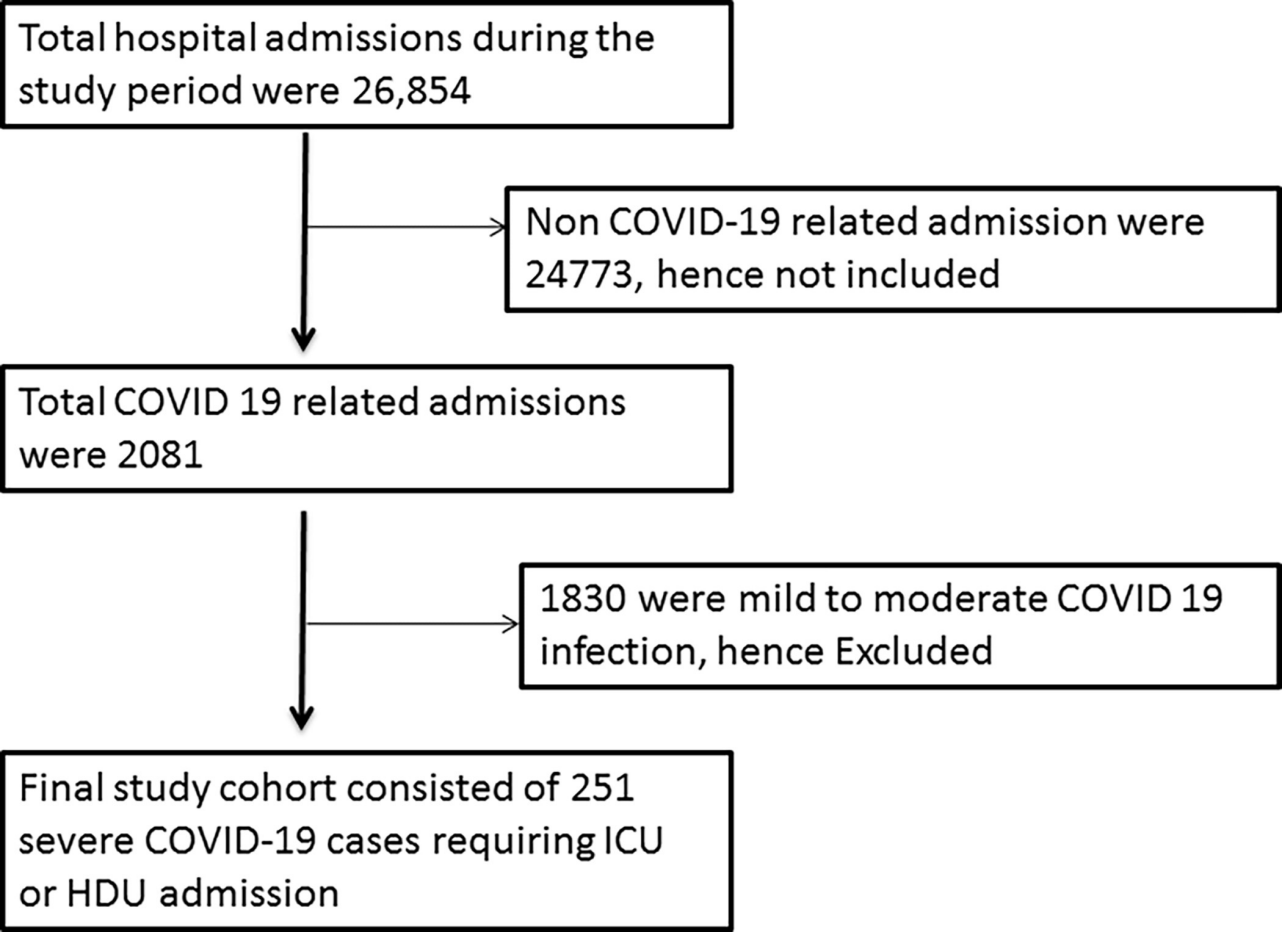
Showing flowchart of the study cohort.

During the study period 26,854 total hospital admissions occurred and among them 2,081 were COVID-19 related admissions. Out of them 251 had severe COIVD-19 infection requiring ICU or HDU admission and formed the study cohort (Fig 1). The mean age of the cohort was 61 ±14.3 years. The study comprised of 169(67.3%) men and 82 women (32.7%). The mean overall survival status in days was 19.84 days with a 95% confidence interval of 16.54 and 23.14 days.

Uni-variate analysis of baseline demographics and co-morbidities among survivors and non survivors are shown in Table 1. Association of Hypothyroidism between the survivors and non-survivors’ groups were found to be statistically significant and noted to be a protective factor (OR: 0.298, CI: 0.108–0.817, p value 0.019) in our study population. Uni-variate analysis of laboratory parameters and its association with survival is shown in Table 2.

**Table 1:** Uni-variate analysis of baseline demographics and survival.

Variables	Categories	Survivors n (%)	Non-Survivors n (%)	OR	95% CI median		p value
					L B	U B	
MuLBSTA	< 8, n = 135	108 (80)	27 (20)	8.211	4.630	14.560	<0.001
	≥ 8, n = 116	38 (32.8)	78 (67.2)				
Age	<60, n = 121	71 (54.2)	60 (45.8)	1.408	0.850	2.333	0.183
	≥ 60, n = 120	75 (62.5)	45 (37.5)				
Multilobar infiltrate	Absent, n = 151	112 (74.2)	39 (25.8)	5.575	3.212	9.675	<0.001
	Present, n = 100	34 (34)	66 (66)				
Bacterial Coinfection	Absent, n = 201	136 (67.7)	65 (32.3)	8.369	3.940	17.777	<0.001
	Present, n = 50	10 (20)	40 (80)				
Smoking	Absent, n = 110	74 (67.3)	36 (32.7)	1.970	1.174	3.305	0.010
	Present, n = 141	72 (51.1)	69 (48.9)				
Systemic Hypertension	Absent, n = 127	73 (57.5)	54 (42.5)	1.058	0.864	3.305	0.823
	Present, n = 124	73 (58.9)	51 (41.1)				
Type 2 DM	Absent, n = 143	88 (61.5)	55 (38.5)	1.379	0.831	2.289	0.213
	Present, n = 108	58 (53.7)	50 (46.3)				
CLD	Absent, n = 227	139(61.2)	88 (38.8)	3.836	1.529	9.624	0.004
	Present, n = 24	7 (29.2)	17 (70.8)				
CKD	Absent, n = 223	140(62.8)	83 (37.2)	6.185	2.410	15.875	<0.001
	Present, n = 28	6 (21.4)	22 (78.6)				
CAD	Absent, n = 209	125(59.8)	84 (40.2)	1.488	0.680	8.059	0.240
	Present, n = 42	21 (50)	21 (50)				
Cancer	Absent, n = 226	138(61.1)	88 (38.9)	3.332	1.380	8.049	0.007
	Present, n = 25	8 (32)	17 (68)				
Hypothyroidism	Absent, n = 225	125(55.6)	100 (44.4)	0.298	0.108	0.817	0.019
	Present, n = 26	21 (80.8)	5 (19.2)				
CVA	Absent, n = 231	135(58.4)	96 (41.6)	1.151	0.459	2.884	0.765
	Present, n = 20	11 (55)	9 (45)				
OAD	Absent, n = 225	131(58.2)	94 (41.8)	1.022	0.449	2.325	0.959
	Present, n = 26	15 (57.7)	11 (42.3)				

**Table 2:** Univariate analysis for laboratory investigations and survival.

Variables	Categories	Survivors n (%)	Non-Survivors n (%)	OR	95% CI		p value
					LB	UB	
Platelet count(10^9^/L)	<150, n = 69	21 (30.4)	48 (69.6)	5.013	2.748	9.142	<0.001
	≥150, n = 182	125(68.7)	57 (31.3)				
ALC(10^9^/L)	≤ 0.8, n = 102	41 (40.2)	61 (59.8)	3.550	2.090	6.030	<0.001
	>0.8, n = 149	105(70.5)	44 (29.5)				
Serum Albumin(g/dL)	<3.5, n = 96	29(30.2)	67 (69.8)	7.611	4.259	13.601	<0.001
	≥3.5, n = 146	112 (76.7)	34 (23.3)				
CRP(mg/ml)	> 100, n = 52	17(32.7)	35 (67.3)	3.819	1.996	7.310	<0.001
	≤ 100, n = 197	128(65)	69 (35)				
LDH9 (units/Liter)	≥ 245, n = 128	52(40.6)	76 (59.4)	6.746	3.364	13.525	<0.001
	< 245 units/Liter, n = 73	60 (82.2)	13 (17.8)				
Ferritin(ng/mL)	≥ 300, n = 121	47(38.8)	74 (61.2)	8.397	4.507	15.643	<0.001
	< 300, n = 114	96 (84.2)	18 (15.8)				
D Dimer(mcg/mL)	≥ 1, n = 117	37(31.6)	80 (68.4)	12.176	6.521	22.736	<0.001
	< 1, n = 126	107 (84.9)	19 (15.1)				
Serum Sodium(mmol/L**)**	< 135, n = 120	52 (43.3)	68 (56.7)	3.378	1.993	5.726	<0.001
	≥ 135, n = 129	93(72.1)	36 (27.9)				

The variables which were showing P value <0.005 were subjected to multivariate analysis.

On multivariate analysis of the individual parameters in the scoring system, Multi lobar infiltrate (OR:3.620, CI:1.972–6.643, p-value <0.001), Absolute lymphocyte count (OR:2.684, CI:1.461–4.931, p-value:0.001), and Bacterial co infection (OR:6.531, CI: 2.877–14.825, p-value:<0.001) were found to be independent risk factors associated with mortality as shown in Table 3. Multivariate cox regression analysis after adjusting all the factors included in our study, showed that platelet counts<150000 (HR: 1.989, CI: 1.239–3.194, p-value: 0.004), Serum LDH ≥ 245(HR:1.908, CI: 1.004–3.579, p-value:0.049), and D Dimer ≥1 (HR:1.895, CI: 1.004–3.579, p-value:0.049) were found to be statistically significant predictors of mortality in COVID patients (Table 3).

**Table 3:** Multivariate analysis—Prognostic factors for mortality on day of admission.

Variable	B	Wald	HR (95% of CI HR)	p value
Platelet count <150x10^9^/L	0.688	8.111	1.989(1.239–3.194)	0.004
Serum albumin < 3.5 g/dL	0.418	2.693	1.519(0.922–2.503)	0.101
Serum LDH ≥ 245	0.646	3.891	1.908(1.004–3.625)	0.049
D Dimer ≥ 1	0.639	3.886	1.895(1.004–3.579)	0.049
Multi-lobar infiltrate (+)	1.286	17.244	3.620(1.972–6.643)	**<0.001**
ALC (≤0.8x 10^9^/L)	0.987	10.129	2.684 (1.461–4.931)	**<0.001**
Bacterial coinfection (+)	1.877	20.131	6.531(2.877–14.825)	**<0.001**
Smoking (+)	0.342	1.210	1.408(0.765–2.590)	0.271
Systemic hypertension (+)	0.108	0.119	0.898(0.487–1.656)	0.730
Age (≥ 60 years)	0.281	0.802	1.325(0.716–2.453)	0.371

On multivariate analysis, Multi lobar infiltrate (OR:3.620, CI:1.972–6.643, p-value <0.001), Absolute lymphocyte count (OR:2.684, CI:1.461–4.931, p-value:0.001), and Bacterial co-infection (OR:6.531, CI: 2.877–14.825, p-value:<0.001) were found to be independent risk factors associated with mortality.

**Fig 2:**
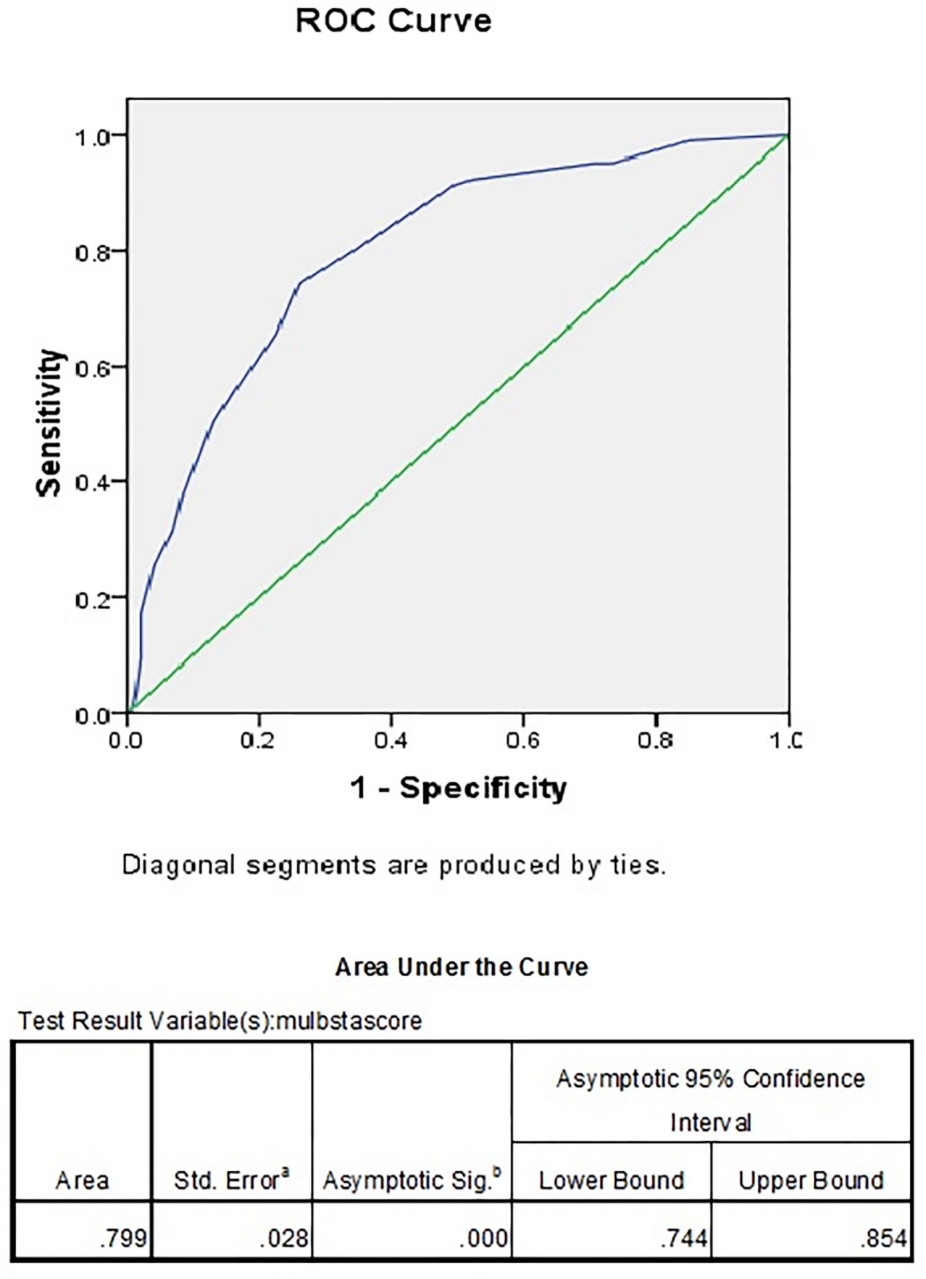
ROC of MuLBSTA score and mortality.

The area under the receiver operating characteristic curve (ROC) of MuLBSTA for predicting mortality at the time of admission was 0.799 (SE 0.028) as shown in Fig 2. The ROC analysis in our study revealed a cut off value for MuLBSTA score to be 8, with a sensitivity of 74.29% and specificity of 73.97% (Fig 2). The association between MuLBSTA score ≥ 8 (OR:8.211, CI: 4.630–14.560, p value <0.001) and mortality was found to be statistically significant with p value of <0.001. MuLBSTA score≥8 had a Positive Predictive value of 67.24%, Negative Predictive value of 80% with an Accuracy of 74.10% in our study.

**Fig 3:**
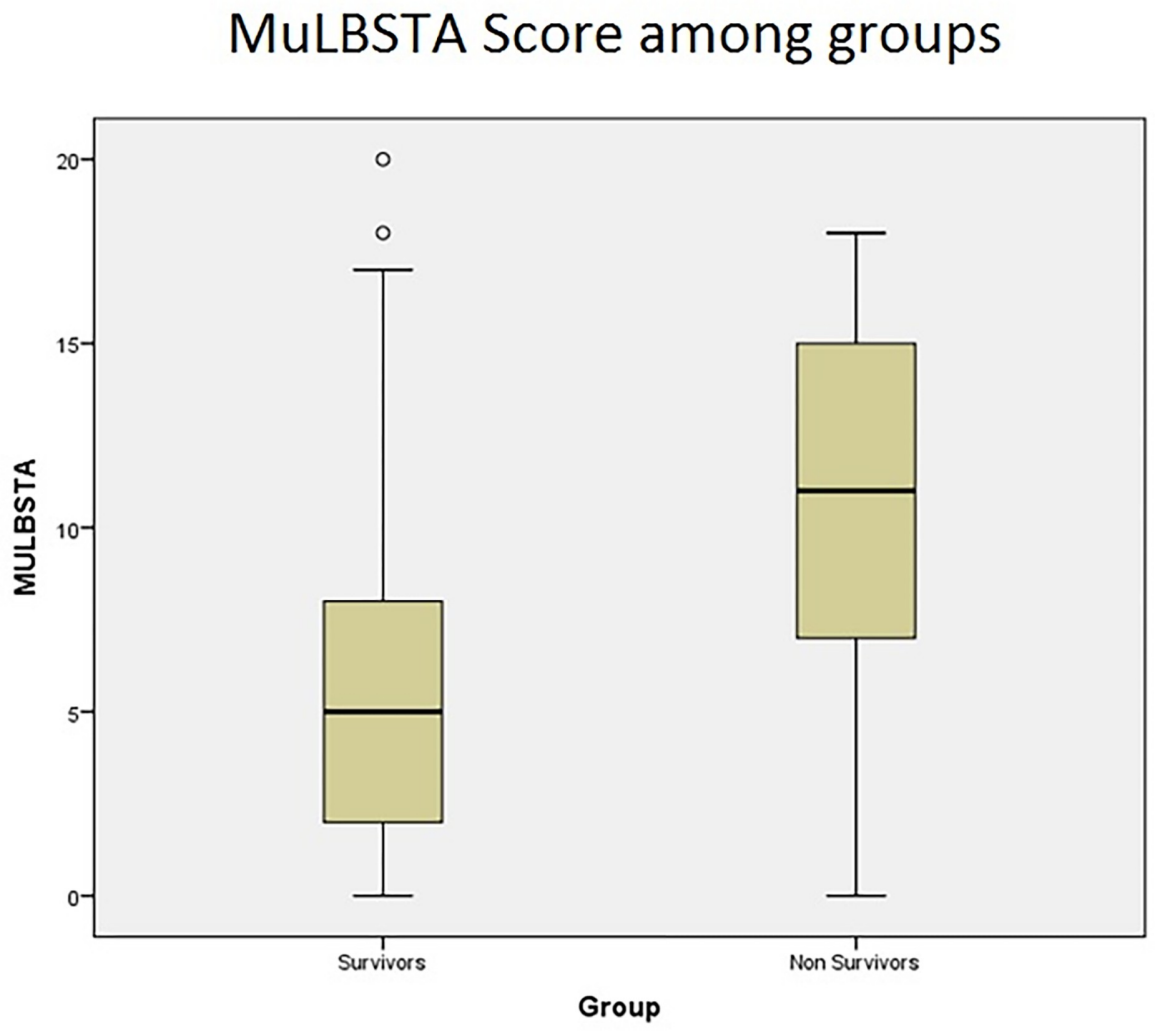
MuLBSTA score among groups.

The median (Q1, Q3) value of MuLBSTA score among survivors was 5 (2–8), and the median (Q1, Q3) value of MuLBSTA score among non-survivors was 11(7–15). The P value was found to be statistically significant (<0.001) as shown in Fig 3.

**Fig 4:**
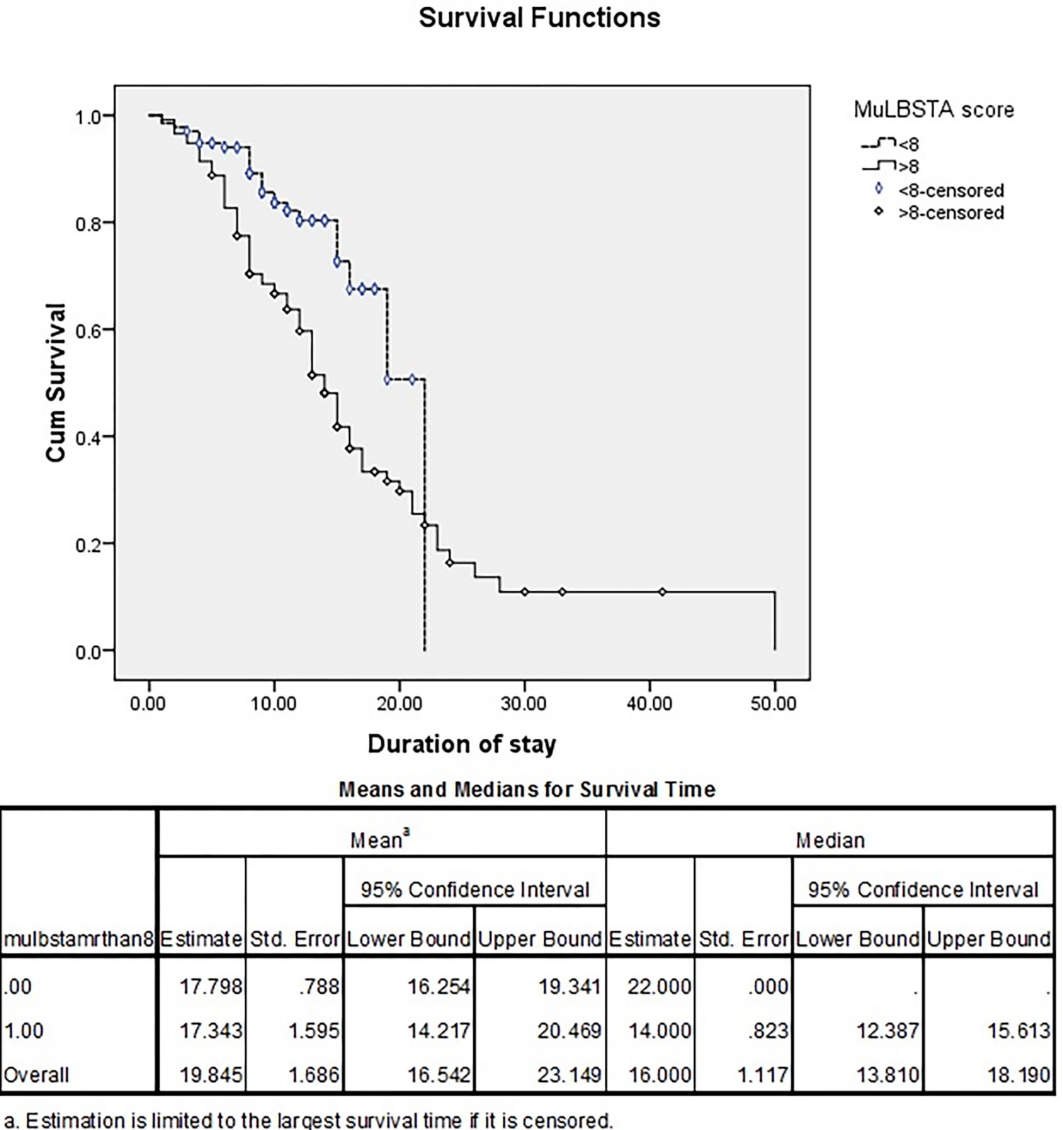
Kaplan-Meier curve of comparison of MuLBSTA score of <8 with >8.

Out of the 116 patients with MuLBSTA score ≥8, 78 expired (67.2%) whereas, out of the 135 patients with MuLBSTA score < 8, 27 expired (20%) during the study period. Kaplan Meier curve was plotted for MuLBSTA score of >8 and <8 days as shown in Fig 4. The median overall survival of patients with MuLBSTA score ≥ 8 was 14 days and in patients with < 8 was 22 days with 95% CI of 12.387 and 15.613 days (P value of <0.001).

### Validation of MuLBSTA score

For validation and deriving modified score linear regression analysis was done and the results are shown in Table 4. The score was modified as per beta coefficient and new scoring system was derived. The new MuLB score of 8 was associated with high risk of poor survival (S2 Table).

**Table 4:** Calculations of the MuLBSTA score for risk stratification in the validation cohort.

Variable	HR(95% CI)	P Value	Beta coefficient by current study	Existing score for MuLBSTA	New proposed scores
Multi lobar infiltrate	4.3(0.07–0.02)-	<0.001	-0.254	5	3
Absolute Lymphocyte count <0.8x10^9^/L	3.29(0.300–0.075)	<0.001	-0.187	4	2
Bacterial Infection	5.45(0.132–0.062)	<0.001	-0.306	4	3
Smoking history	0.646(0.059–0.030)	0.519	-0.036	3	0
Systemic hypertension	0.129(0.101–0.116	0.897	-0.007	2	0
Age >60 years	1.16(0.087–0.022)	0.247	-0.065	2	1

## Discussion

COVID-19 virus has thrown up a multitude of challenges since its first detection. One of the foremost challenges has been the identification of at-risk individuals and appropriate targeting of resources for effective management so as to limit the accompanying mortality. Even though the condition has many similarities with other viral pneumonia’s, we are yet to identify a definite tool to ascertain the disease’s outcome immediately upon infection. It may be noted that the association of various risk factors assists in better prediction of the worse outcomes [8].

Out of t 251 patients, 169 were men and 82 women with a mean age group of 61 years and the male gender was found to be a risk factor associated with mortality. It is well known fact that male gender is an independent risk factor of mortality as found in many studies earlier [8, 9].

Although, the virus infects people of all ages alike, poorer outcomes have been noted in older age groups, and especially those persons above sixty years of age. However, in our study age more than sixty years was not associated with mortality. Patients with smoking history were more prone to die and this must probably be because tobacco smoking likely alters the expression of “angiotensin converting enzyme receptors” making it more likely to be associated with mortality [10]. It is also pertinent to mention that the virus infected individuals are more likely to contract secondary bacterial or fungal co infections resulting in many individuals succumbing to the viral illness [11].

Most patients in our study with multi lobar infiltrates on the chest X-ray and/or lymphopenia during the time of admission had worse outcomes; the finding similar to previously published study [12].

It has been proven by various studies that DM, CKD, CLD, CAD and cancer are comorbidities associated with poor outcome in COVID-19 infection [13–22]. In current study, CLD (OR:3.836, CI:1.529–9.624, p value 0.004), CKD (OR:6.185, CI:2.410–15.875, p value <0.001), Cancer (OR:3.332, CI:1.380–8.049, p value 0.007), were found to be independent risk factors associated with mortality while the association of Systemic hypertension(OR:1.058(0.864–3.305), Type 2 DM (OR:1.379, CI:0.831–2.289, p-value:0.213), CAD (OR:1.488, CI:0.680–8.059, p value 0.240), OAD(OR:1.022, CI:0.449–2.325, p value 0.959), and CVA(OR:1.151, CI: 0.459–2.884, p value 0.765) were not found to be statistically significant. We do not have any explanation for the above finding other than that the present study included seriously ill patients with more severe disease and DM was just one of the variables.

Previous studies have shown that there is no impact of Hypothyroidism on COVID 19 mortality [23]. However, in the present study, hypothyroidism was found to be a protective factor (OR: 0.298, CI: 0.108–0.817, p value 0.019) against mortality. The results should be taken with caution as only 26 patients had hypothyroidism and more studies are needed to confirm our finding.

The laboratory parameters that were obtained at the time of admission were analyzed and it was observed that Serum Albumin <3.5g/dl (OR:7.611, CI:4.259–13.601, p value <0.001), CRP > 100 mg/mL (OR:3.819, CI:1.996–7.310, p value <0.001), LDH ≥245 units/Litre (OR:6.746, CI:3.364–13.525, p value <0.001), Serum Ferritin≥300 ng/mL (OR:8.397, CI:4.507–15.643, p value <0.001), D Dimer ≥1 mcg/mL (OR:12.176, CI:6.521–22.736, p value <0.001), Serum sodium<135 **mmol/L** (OR:3.378, CI:1.993–5.726, p value <0.001), Platelet count<150x 10^9^/L (OR:5.013, CI:2.748–9.142, p value <0.001) were found to be independent risk factor associated with mortality. On multi-variable cox regression analysis after adjusting for all the factors that were obtained during the time of admission in our study, platelet counts<15010^9^/L (HR: 1.989, CI: 1.239–3.194, p-value: 0.004), Serum LDH ≥ 245 units/Litre (HR:1.908, CI: 1.004–3.579, p-value:0.049), and D Dimer ≥1 mcg/mL (HR:1.895, CI: 1.004–3.579, p-value:0.049) were found to be statistically significant predictors of mortality in patients with COVID 19 infection. The finding was similar to other studies [24–33]. Hypoalbuminemia, elevated CRP, and elevated d-dimer and Ferritin are associated with adverse outcome among COVID-19 as previously shown in other studies [2, 24–34]. Many of the aforementioned factors have been taken into account in the MuLBSTA scoring system [34, 35]. The overall survival among the patients with a MuLBSTA score more than eight was significantly less in the current study.

MuLBSTA scoring system was proven to be a good model for predicting mortality and risk stratification of the patients in a study by Rong Xu et al. [36] Prognosis scores routinely used for CAP (PSI and CURB-65) were good predictors for mortality in patients with COVID-19 CAP but not for need of hospitalization or ICU admission. Study by Garcia Clement MM. et al. showed that MuLBSTA score was better at predicting the need for ICU admissions than other prognostic scores such as PSI‑PORT and CURB‑65 [35–37].

We also did validation of MuLBSTA score in the study cohort and derived the new score for each variable as shown in S2 Table. As per the new MuLB score, the score of 8 is associated with poor survival. Unlike the single-risk indicator found in previous studies, our scoring system combined a variety of clinical and laboratory indicators and comprehensively and accurately predicted patient’s survival. Out of the 116 patients with MuLBSTA score ≥8, 78 expired (67.2%) whereas, out of the 135 patients with MuLBSTA score < 8, 27 expired (20%) during the study period. However, the new score needs validation by other studies to determine the reproducibility of the results.

### Strength and limitations

The strength of our study was that we were able to include two hundred fifty-one patients which attributed to a good sample size.

The limitations of our study are that it was single center study and the intrinsic bias associated while collecting some data retrospectively could not be avoided. The MuLBSTA score was used to predict ninety-day mortality in all the previous studies and our study was limited to following up patients only for a period of 28 days. The laboratory values of patients who developed the viral infection as a part of hospital acquired infection was taken during the date of positivity and not at the time of admission. The new MuLB score is very promising but it needs further validation by other studies.

## Conclusion

Our study validated the MuLBSTA score with ROC analysis and score of ≥ 8 was dependable tool to assess 28-day mortality in seriously ill COVID-19 positive patients. We also derived the new MuLB score which was better score than existing score to identify the high risk COVID-19 patients. It was also noted that high levels of C-reactive protein, Serum Ferritin, Serum Lactate dehydrogenase, D Dimer and low levels of serum albumin, sodium, platelet and absolute lymphocyte counts were associated with increased mortality. Co -morbidities such as CKD, CLD and cancer were also associated with poor outcome. It may hence be concluded that such a tool may help primary care medical doctors in reallocating patients to centers with better care and facilities and as a consequent thereof even aid in containing the disease and its mortality rates.

## Supporting information

S1 Table The MuLBSTA score. (DOCX)

S2 Table Best proposed modified model: MuLBA or MuLB score. (DOCX)

S1 Data Final data to be kept in public domain. (XLSX)

S2 Data Data of hypothyroid patients. (XLSX)
