# Supplementary material for: Validation of MuLBSTA score to derive modified MuLB score as mortality risk prediction in COVID-19 infection
Source: PLOS Glob Public Health. 2022 Aug 1;2(8):e0000511. doi: 10.1371/journal.pgph.0000511 (PMC10021136; doi:10.1371/journal.pgph.0000511)
Supplement: S1 Table — (DOCX) [file pgph.0000511.s001.docx]

S1 Table: The MuLBSTA score ^5-6^

| **MuLBSTA** | **YES** | **NO** |
| --- | --- | --- |
| Multilobular infiltrate | +5 | 0 |
| Absolute Lymphocyte count <0.8x10^9^ | +4 | 0 |
| Bacterial coinfection | +4 | 0 |
| Smoking History | Active smoker +3 | 0 |
|  | Prior smoker +2 |  |
| Systemic hypertension | +2 | 0 |
| Age >60yrs | +2 | 0 |
