# Supplementary material for: Validation of MuLBSTA score to derive modified MuLB score as mortality risk prediction in COVID-19 infection
Source: PLOS Glob Public Health. 2022 Aug 1;2(8):e0000511. doi: 10.1371/journal.pgph.0000511 (PMC10021136; doi:10.1371/journal.pgph.0000511)
Supplement: S2 Table — (DOCX) [file pgph.0000511.s002.docx]

**S2 Table: Best proposed modified model: MuLBA or MuLB score.**

| Variables | Score | Final Score |
| --- | --- | --- |
| Mu+L+B+A | 3+2+3+1 | 9 |
| Mu+L+B | 3+2+3 | 8 |

Mu: Multi lobar infiltrate, L : Lymphocyte <0.8x10^9^/L, B: bacterial co-infection,

A: Age >60 year
